# Suicide Mortality Among Young People in Brazil: A National Time-Series Analysis, 2000-2023

**DOI:** 10.7759/cureus.114601

**Published:** 2026-08-16

**Authors:** Catherine R Vitoi, Troy R Carneiro Filho, Iago F Rego Brito, Laís Teles Corrêa M De Castro

**Affiliations:** 1 Faculty of Medicine, Centro Universitário de Brasília (CEUB), Brasília, BRA; 2 Medical School, Instituto Master de Ensino Presidente Antônio Carlos (IMEPAC), Araguari, BRA; 3 Medical School, Centro Universitário FIPMoc, Montes Claros, BRA; 4 Faculty of Medicine, Centro Universitário de Brasília (CEUB), Brasilia, BRA

**Keywords:** adolescents, brazil, health disparities, mortality, suicide, time-series analysis, young adults

## Abstract

Background

Suicide mortality among young people has increased in Brazil, but prior studies have used heterogeneous age-group definitions and may have underestimated the burden by relying only on deaths coded as intentional self-harm.

Methods

This nationwide ecological time-series study used data from the Brazilian Mortality Information System and official population estimates. All deaths among individuals aged 10-29 years from 2000 to 2023 were included. Analyses were stratified by sex, age group, and geographic region. Suicide deaths were defined using the International Statistical Classification of Diseases and Related Health Problems, Tenth Revision (ICD-10) codes X60-X84. Age-standardized mortality rates were calculated using the 2022 Brazilian population. Trends were assessed using joinpoint and negative binomial regression models, with a sensitivity analysis including deaths of undetermined intent.

Results

Between 2000 and 2023, 77,012 suicide deaths occurred among individuals aged 10-29 years. The age-standardized mortality rate increased from 3.68 to 7.37 per 100,000. Rates rose in both males and females and across all age groups, with faster increases after 2015. The 10- to 14-year group showed the largest relative increase, while mortality among those aged 25-29 years accelerated after 2018. Including deaths of undetermined intent increased overall estimates but did not change trend patterns.

Conclusions

Suicide mortality among Brazilian individuals aged 10-29 years doubled between 2000 and 2023, with acceleration in recent years. The findings highlight important heterogeneity across age groups and regions, supporting the need for more granular surveillance and targeted prevention strategies.

## Introduction

Suicide is among the leading causes of death in young people worldwide, and the mental disorders that most often precede it reach their greatest population burden in adolescence [1]. In Brazil, as deaths from infectious and perinatal causes have fallen, injury and self-inflicted death have come to dominate the mortality profile of young people, giving the epidemiology of youth suicide a prominence it did not previously hold [2].

National estimates point consistently in one direction. Although the age-standardized suicide rate in Brazil fell between 1990 and 2019, the absolute number of deaths rose substantially, and the only age group in which the rate increased was the youngest [3]. Subsequent ecological studies have documented rising suicide rates among Brazilian adolescents, with the steepest increases among girls in early adolescence [4], a comparable upward trend extending into young adulthood [5], and a growing loss of potential years of life across affected states [6]. On present trajectories, most Brazilian states are not expected to meet international targets for suicide reduction [7].

Interpretation of this evidence is limited in two respects. First, previous Brazilian studies have divided young people into inconsistent age bands so that no single analysis describes the continuous span from 10 to 29 years within one framework, obscuring the early rise now emerging in the youngest girls. Second, the underlying cause of death for external causes is incompletely specified in national mortality data, with a substantial proportion of deaths assigned to ill-defined codes or to undetermined intent, so that analyses confined to explicitly coded self-harm are liable to underestimate the true burden [8].

We therefore examined trends in suicide mortality among people aged 10-29 years in Brazil from 2000 to 2023, stratified by sex, narrow age group, and geographic region, and quantified the influence of misclassified intent by comparing a primary case definition of intentional self-harm with a prespecified expanded definition that additionally included deaths of undetermined intent and sequelae of self-harm.

## Materials and methods

Study design and setting

We conducted a nationwide, population-based ecological time-series study of suicide mortality among young people in Brazil from 2000 through 2023. The unit of analysis was the population aggregate rather than the individual. We followed established reporting guidance for observational studies using routinely collected health data.

Data sources

Mortality data were obtained from the Mortality Information System (Sistema de Informações sobre Mortalidade, SIM) of the Brazilian Ministry of Health, a national, publicly available registry of death certificates coded according to the International Statistical Classification of Diseases and Related Health Problems, Tenth Revision (ICD-10). SIM provides continuous national coverage throughout the study period. The temporal variable was the calendar year of death.

Population denominators by sex, age group, and region were obtained from the Brazilian Institute of Geography and Statistics using the 2000, 2010, and 2022 Demographic Censuses and official intercensal population estimates and are publicly available [9]. Records with missing age (0% of registrations aged 10-29 years in every study year) or missing sex (≤0.0002% in any single year) were excluded from the corresponding stratified analyses.

Study population and case definitions

The study population comprised all residents of Brazil aged 10-29 years. To characterize age-specific patterns across adolescence and young adulthood, participants were stratified into four age groups: 10-14, 15-19, 20-24, and 25-29 years. Analyses were also stratified by sex.

The primary outcome was death from intentional self-harm, defined by an underlying cause of death coded as ICD-10 X60-X84 [10]. A prespecified expanded case definition was also examined in sensitivity analyses, adding deaths coded to poisoning of undetermined intent (ICD-10 Y10-Y19) and sequelae of intentional self-harm of undetermined intent (ICD-10 Y87.0) to the primary definition to assess the influence of misclassified intent on the observed trends.

Statistical analysis

Crude mortality rates were calculated per 100,000 population for each calendar year, sex, age group, and geographic region. Age-standardized rates were calculated by the direct method using the age distribution of the 2022 Brazilian Census population as the standard, applied uniformly across all years and geographic regions.

Temporal trends in rates were estimated by segmented log-linear regression of the natural logarithm of the rate on calendar year, fitted by ordinary least squares. The number and location of breakpoints (0 to 2 per series) were selected by minimizing the Bayesian information criterion. Annual percentage changes were estimated for each segment and average annual percentage changes for the entire study period, with 95% CIs, and classified as increasing, decreasing, or stable using a two-sided significance threshold of P < 0.05.

Comparisons across sex, age group, and region, and all sensitivity analyses, modeled annual death counts directly using generalized linear regression with a log link and the natural logarithm of the population as an offset. Overdispersion was assessed for each series using the Pearson chi-square statistic divided by residual degrees of freedom; a ratio greater than 1.5 was taken as evidence of overdispersion. This threshold was exceeded in every analyzed series, and negative binomial regression was therefore used in place of Poisson regression throughout, with model choice confirmed by the Akaike information criterion.

The COVID-19 period was assessed by interrupted time-series analysis, with 2020 prespecified as the interruption point:

$\log\left\{E(Y_t)\right\} = \beta_0 + \beta_1 T_t + \beta_2 I_t + \beta_3\left(T_t \times I_t\right) + \log(P_t),$

where It indicates calendar years from 2020 onward, β2 estimates the immediate level change, and β3 the change in slope thereafter. As a sensitivity analysis, the log-linear trend models described above were refitted after excluding the years 2020 through 2022 to assess whether pandemic-period observations altered the estimated annual percentage change in any series.

Analyses were performed for the overall population and separately by sex, age group, and geographic region, under both the primary and expanded case definitions.

Ethics

The study used only secondary, aggregated, anonymized data in the public domain and did not permit identification of individuals. Under Resolution 510/2016 of the Brazilian National Health Council, research using this type of publicly available aggregated data is exempt from research ethics committee review.

## Results

Between 2000 and 2023, 77,012 deaths from intentional self-harm were registered among Brazilian residents aged 10-29 years. Of these deaths, 59,466 (77.2%) occurred among males and 17,543 among females; three deaths could not be assigned to a sex category. The annual number of deaths increased from 2,225 in 2000 to 4,655 in 2023 (Table 1).

**Table 1:** Deaths and mortality rates from suicide (ICD-10 X60-X84), ages 10-29 years, Brazil and geographic regions (2000 and 2023). * Rates per 100,000 population, ages 10-29 years. Age-standardized rates were directly standardized to the age distribution of the Brazilian population in 2022 (4 bands: 10-14, 15-19, 20-24, and 25-29 years), applied uniformly to all strata, regions, and years. ICD-10, International Statistical Classification of Diseases and Related Health Problems, Tenth Revision

Area	Deaths (2000)	Crude rate (2000*)	Age-standardized rate (2000*)	Deaths (2023)	Crude rate (2023*)	Age-standardized rate (2023*)
Brazil - Overall	2,225	3.41	3.68	4,655	7.37	7.37
Brazil - Men	1,690	5.17	5.70	3,545	11.06	11.10
Brazil - Women	535	1.64	1.70	1,110	3.57	3.56
North	196	3.60	3.91	634	9.36	9.48
Northeast	416	2.14	2.37	1,187	6.74	6.87
Southeast	753	2.82	2.99	1,465	5.93	5.90
South	555	6.14	6.57	807	9.69	9.56
Central-West	305	6.56	6.78	562	11.09	11.13

Age-standardized suicide mortality doubled during the study period, increasing from 3.68 to 7.37 deaths per 100,000 population. Among males, mortality increased from 5.70 to 11.10 per 100,000, while among females it increased from 1.70 to 3.56 per 100,000. The male-to-female mortality rate ratio decreased modestly from 3.35 in 2000 to 3.12 in 2023.

Crude and age-standardized rates were similar in all strata. Across the analyzed series, the largest difference between crude and standardized estimates of annual change was 0.48 percentage points.

National and sex-specific trends

**Figure 1:**
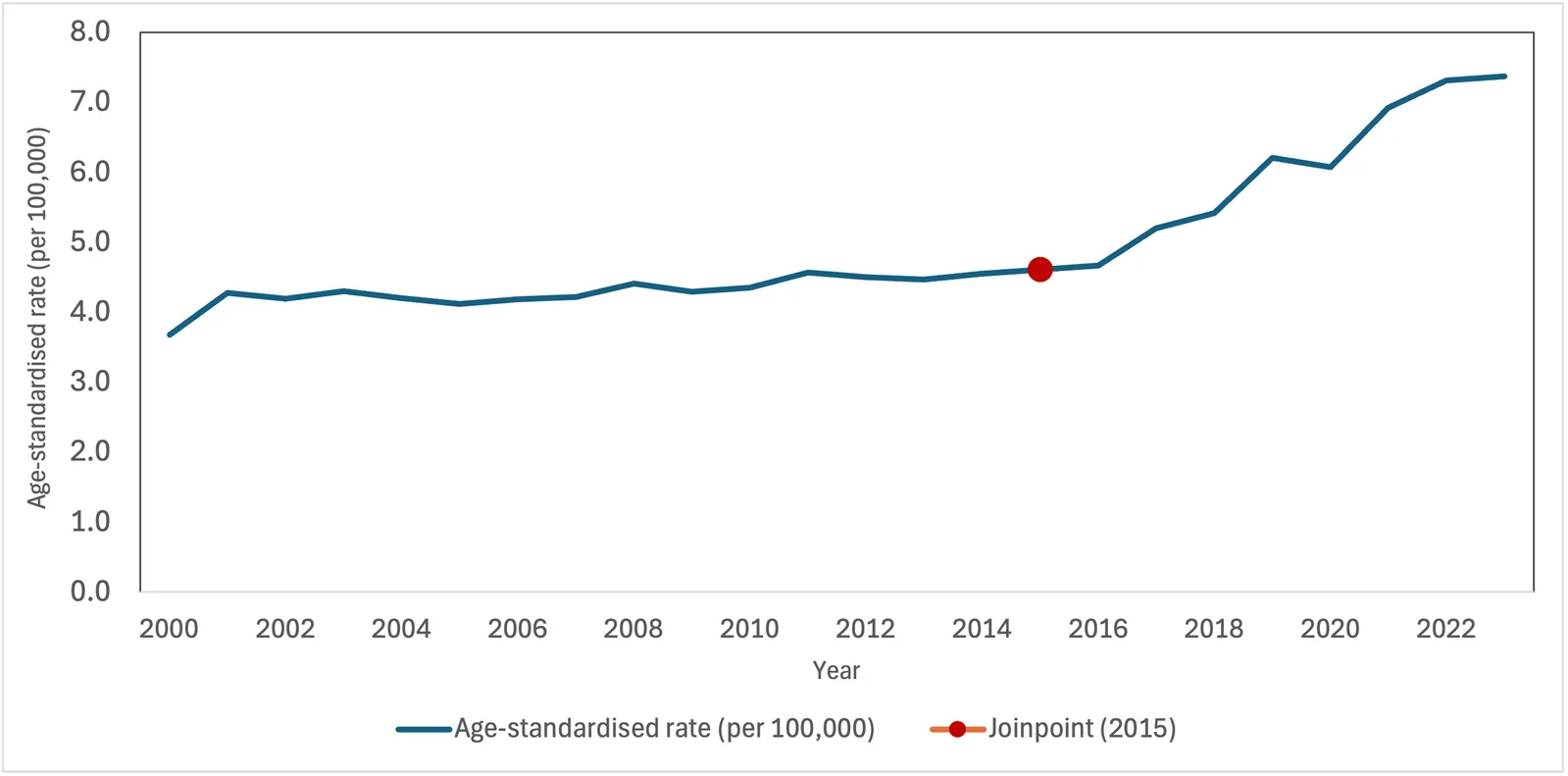
Age-standardized suicide mortality among people aged 10-29 years, Brazil (2000-2023). Rates are per 100,000 population, standardized by the direct method to the age structure of the 2022 Brazilian Census. The red marker indicates the joinpoint identified at 2015, before which the annual percentage change was 0.86% (P < 0.001) and after which it was 6.58% (P < 0.001); the average annual percentage change over the full period was 2.72%.

Joinpoint regression identified a single breakpoint in the national series in 2015. The age-standardized rate increased by 0.86% per year (95% CI, 0.51%-1.21%; P < 0.001) from 2000 through 2015, accelerating to 6.58% per year (95% CI, 5.82%-7.34%; P < 0.001) thereafter. The average annual percentage change from 2000 through 2023 was 2.72% (Table 2, Figure 1).

**Table 2:** Joinpoint analysis of age-standardized mortality rates from suicide (ICD-10 X60-X84), ages 10-29 years, Brazil (2000-2023). APC and 95% CIs were estimated by segmented (piecewise) log-linear regression of the natural logarithm of the age-standardized mortality rate on calendar year. The slope of each segment was estimated by ordinary least squares, and P-values and 95% CIs for the slope were derived from a two-sided t test; APC was calculated as 100 × (e^slope − 1). A trend was classified as increasing or decreasing if P < 0.05 (direction determined by the sign of the APC) and as stable otherwise. AAPC, average annual percent change; APC, annual percent change; ICD-10, International Statistical Classification of Diseases and Related Health Problems, Tenth Revision

Sex	Period	APC (95% CI)	P-value	Trend	AAPC (2000-2023)
Brazil - Overall	2000-2015	0.86%/year (0.51% to 1.21%)	<0.001	Increasing	2.72%/year
	2015-2023	6.58%/year (5.82% to 7.34%)	<0.001	Increasing	
Brazil - Men	2000-2015	0.92%/year (0.56% to 1.28%)	<0.001	Increasing	2.49%/year
	2015-2023	5.71%/year (4.94% to 6.49%)	<0.001	Increasing	
Brazil - Women	2000-2015	0.28%/year (-0.17% to 0.73%)	0.207	Stable	2.98%/year
	2015-2021	10.76%/year (9.38% to 12.16%)	<0.001	Increasing	
	2021-2023	1.06%/year (-4.11% to 6.52%)	0.679	Stable	

The trajectory among males was similar, with the same breakpoint in 2015: an annual percentage change of 0.92% (95% CI, 0.56%-1.28%; P < 0.001) before 2015, accelerating to 5.71% (95% CI, 4.94%-6.49%; P < 0.001) thereafter, with an average annual percentage change of 2.49% over the full period.

Among females, mortality remained approximately stable through 2015, with an annual percentage change of 0.28% (95% CI, −0.17% to 0.73%; P = 0.21). Mortality subsequently increased by 10.76% per year from 2015 through 2021 (95% CI, 9.38%-12.16%; P < 0.001) and was stable from 2021 through 2023, with an annual percentage change of 1.06% (95% CI, −4.11% to 6.52%; P = 0.68).

In the negative binomial regression, the mean annual change over the full study period was 2.37% among males (95% CI, 1.96%-2.79%) and 2.79% among females (95% CI, 2.35%-3.23%). The year-by-sex interaction was not statistically significant (likelihood ratio χ² = 1.94; df = 1; P = 0.16).

Age-specific trends

**Figure 2:**
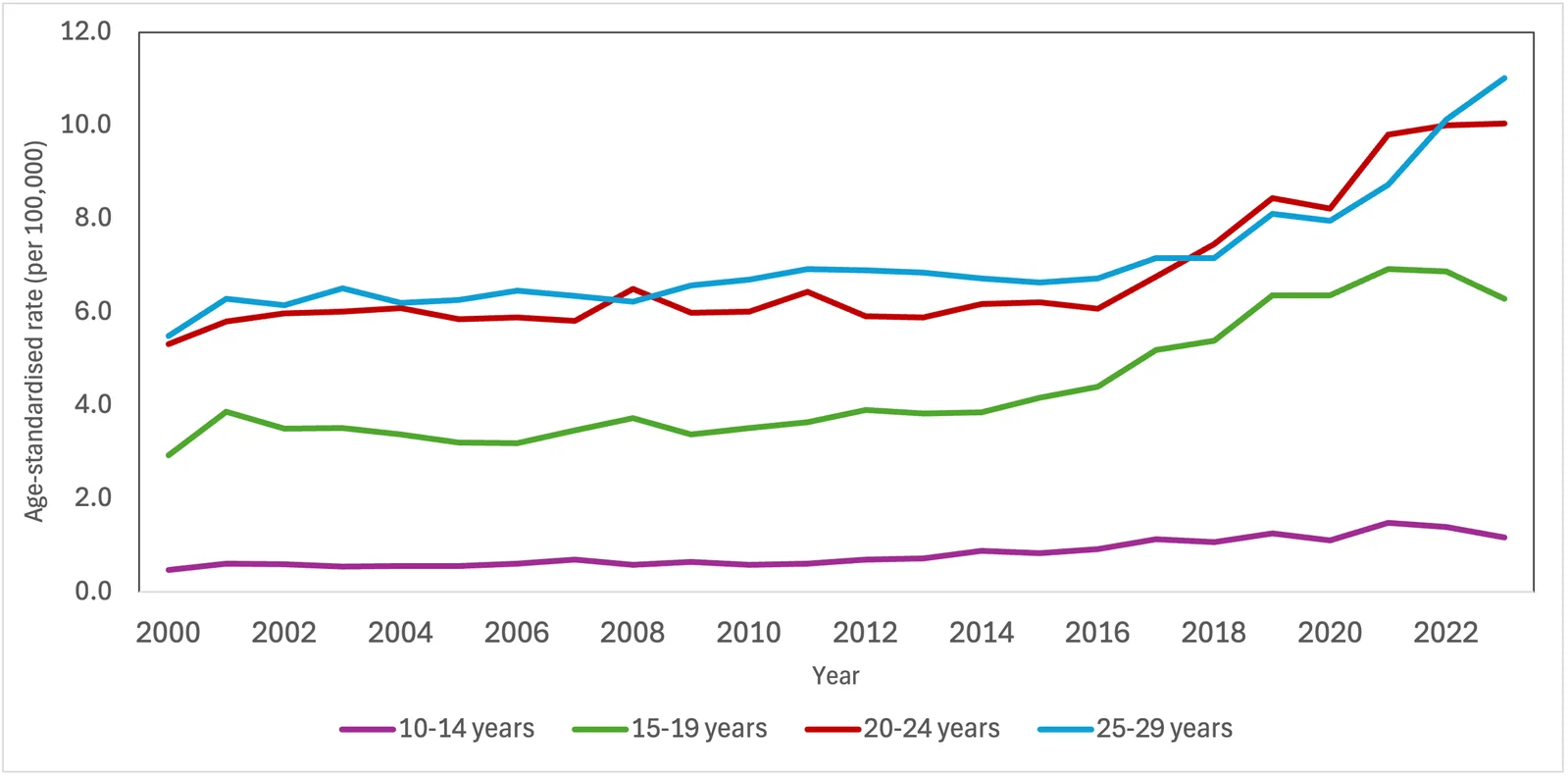
Age-specific suicide mortality by five-year age group, Brazil (2000-2023). Rates are age-specific mortality rates per 100,000 population within each five-year age group.

Temporal trends differed significantly across the four age groups (likelihood ratio χ² = 25.07; df = 3; P < 0.001). Across the full study period, the mean annual increase was greatest among individuals aged 10-14 years, at 3.68% per year (95% CI, 3.03%-4.34%). The magnitude of the annual increase declined progressively with age and was lowest among individuals aged 25-29 years, at 1.80% per year (95% CI, 1.28%-2.33%) (Figure 2).

Among individuals aged 10-14 years, mortality increased by 8.63% per year beginning in 2011 and declined by 8.57% per year after 2021 (P = 0.011). Among those aged 15-19 years, mortality increased by 8.88% per year beginning in 2013 and declined by 6.65% per year after 2021 (P = 0.006).

Among individuals aged 20-24 years, the most recent segment showed no statistically significant change, with an annual percentage change of 2.37% (P = 0.13). Among individuals aged 25-29 years, mortality continued to increase and accelerated after 2018, with an annual percentage change of 7.83% (P < 0.001).

Regional trends

**Figure 3:**
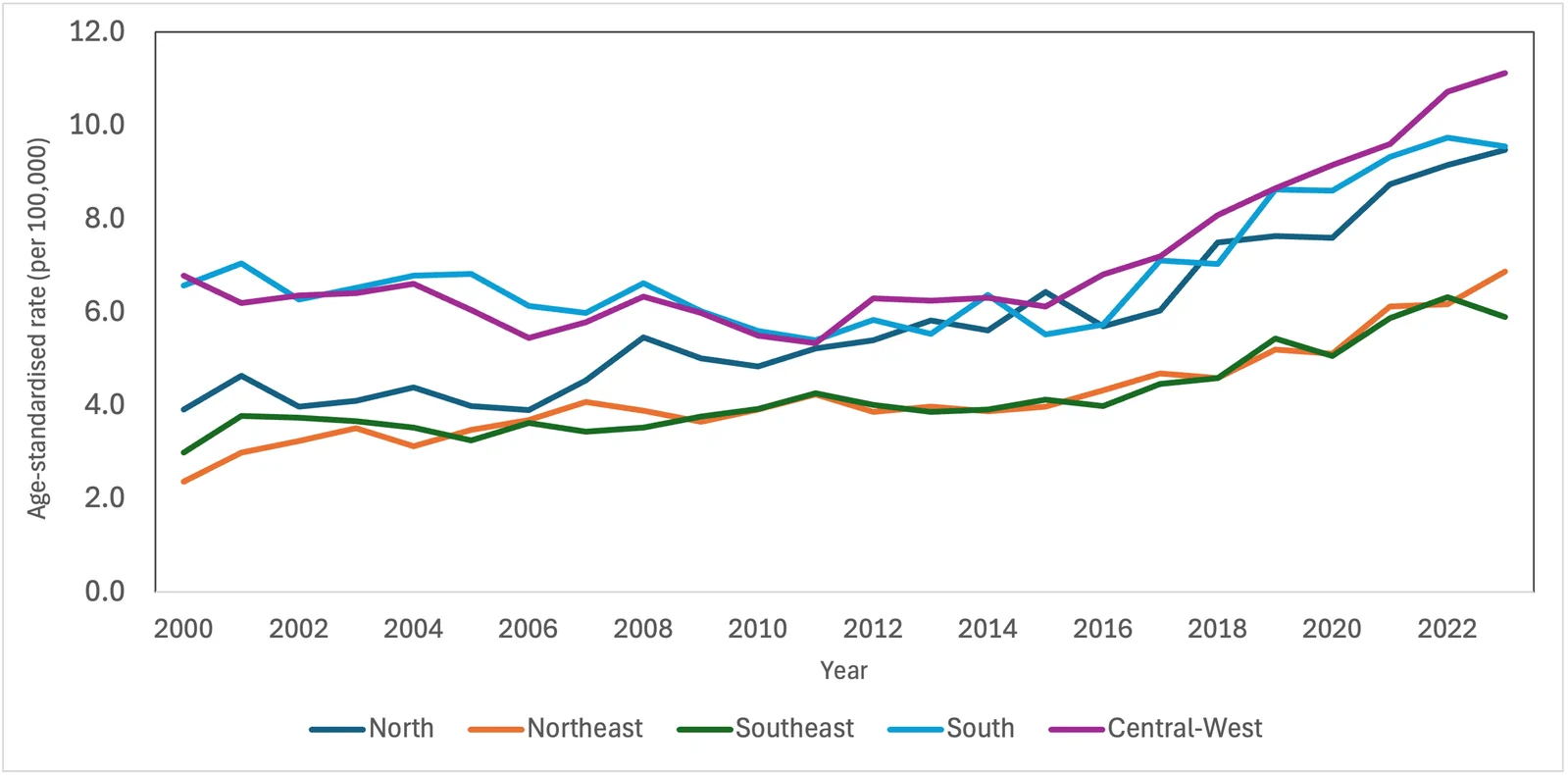
Age-standardized suicide mortality among people aged 10-29 years by region, Brazil (2000-2023). Rates are per 100,000 population, standardized by the direct method to the age structure of the 2022 Brazilian Census, using a single national standard population for all regions so that comparisons are not confounded by regional differences in age composition.

In 2023, age-standardized suicide mortality was highest in the Central-West region, at 11.13 deaths per 100,000 population, followed by the South at 9.56, the North at 9.48, and the Southeast at 5.90 per 100,000 (Figure 3).

Regional trends differed substantially over the study period. The fastest mean annual increase occurred in the North, at 3.80% per year, followed by the Northeast at 3.22%, the Southeast at 2.47%, the Central-West at 2.19%, and the South at 1.40% per year (likelihood-ratio test for region × year interaction: 55.5; df = 4; P < 0.0001).

Although the South and Central-West had comparatively high mortality rates at the beginning of the study period, the more rapid increases in the North and Northeast progressively reduced the differences between regions.

Sensitivity analysis: expanded case definition

Adding deaths from poisoning of undetermined intent (ICD-10 Y10-Y19) and sequelae of self-harm of undetermined intent (Y87.0) to the primary definition increased the total number of deaths from 77,012 to 80,986, an increase ranging from 2.9% among males to 7.1% among females across the eight analyzed series (Table 3). Under the expanded definition, the annual percentage change differed from the primary estimate by no more than 0.31 percentage points in any series, and the direction and statistical significance of the trend were unchanged throughout.

**Table 3:** Sensitivity analysis of suicide mortality estimates using primary and expanded case definitions, Brazil (2000-2023). The primary case definition included deaths from intentional self-harm classified according to the ICD-10 codes X60-X84. The expanded definition additionally included deaths of undetermined intent (Y10-Y19) and sequelae of intentional self-harm (Y87.0). Percentage increase in burden represents the relative increase in the number of deaths under the expanded definition compared with the primary definition. The difference is calculated as the expanded AAPC minus the primary AAPC and is expressed in percentage points. P-values refer to the statistical significance of the temporal trend estimates. AAPC, average annual percentage change; ICD-10, International Statistical Classification of Diseases and Related Health Problems, Tenth Revision

Area	Primary definition (n)	Additional deaths (n)	Expanded definition (n)	Increase in burden (%)	Primary AAPC (%/y)	P-value	Expanded AAPC (%/y)	P-value	Difference (percentage points)
Brazil - Overall	77,012	3,974	80,986	5.16%	2.86	<0.001	2.72	<0.001	-0.14
Brazil - Men	59,466	2,724	62,190	4.58%	2.75	<0.001	2.65	<0.001	-0.09
Brazil - Women	17,543	1,250	18,793	7.13%	3.09	<0.001	2.78	<0.001	-0.31
North	8,708	245	8,953	2.81%	4.16	<0.001	4.04	<0.001	-0.12
Northeast	18,583	1,250	19,833	6.73%	3.54	<0.001	3.23	<0.001	-0.31
Southeast	26,558	1,707	28,265	6.43%	2.8	<0.001	2.68	<0.001	-0.13
South	14,677	524	15,201	3.57%	1.84	<0.001	1.84	<0.001	-0.01
Central-West	8,486	249	8,735	2.93%	2.37	<0.001	2.28	<0.001	-0.09

COVID-19 pandemic period

In the interrupted time-series analysis, 2020 was prespecified as the interruption point. Before 2020, suicide mortality increased by 1.71% per year (95% CI, 1.34%-2.08%; P < 0.001). An immediate upward level change of 23.31% occurred in 2020 (95% CI, 10.77%-37.27%; P < 0.001). There was no statistically significant change in the post-2020 slope (P = 0.26).

Excluding the years 2020 through 2022 reduced the estimated annual percentage change in all analyzed series by 0.20-0.71 percentage points. At the national level, the estimated annual increase declined from 2.47% to 2.03% per year, with both estimates remaining statistically significant (P < 0.001).

## Discussion

Suicide mortality among Brazilians aged 10-29 years doubled between 2000 and 2023, from 3.68 to 7.37 per 100,000, with acceleration after 2015. The increase predated the COVID-19 pandemic and remained significant when 2020 through 2022 were excluded. Males accounted for more than three-quarters of the 77,012 deaths, although the average long-term rate of increase did not differ significantly by sex.

Direct comparison with previous Brazilian studies is difficult because earlier analyses used different age groupings and rarely covered the entire 10- to 29-year range [4-6]. By examining four five-year age groups within the same analytical framework, the present study showed that suicide mortality did not follow a uniform trajectory across adolescence and young adulthood. The greatest mean annual increase occurred among individuals aged 10-14 years. In the most recent period, however, continued growth was observed only among those aged 25-29 years. The national increase was therefore not confined to adolescence. Previous age-period-cohort studies have reported higher suicide risk in more recent Brazilian birth cohorts, although most excluded individuals younger than 20 years [11]. The declines observed after 2021 among those aged 10-19 years may represent a recent change in direction, but these estimates were derived from short terminal joinpoint segments and should be interpreted as exploratory.

Male mortality remained approximately three times higher than female mortality throughout the study period. The female series, however, accelerated sharply between 2015 and 2021, consistent with previous findings among girls aged 10-14 years [4]. Because the year-by-sex interaction was not significant, the data do not indicate different long-term rates of increase between males and females. They suggest, instead, that the acceleration occurred at different times.

Regional convergence resulted from faster increases in the North and Northeast rather than declines in regions where mortality had been highest. Earlier spatial and regional age-period-cohort analyses documented heterogeneity without distinguishing current mortality levels from long-term change [11,12]. In 2023, mortality was highest in the Central-West, whereas growth was fastest in the North. Socioeconomic indicators have been associated with adolescent suicide rates [13], and a national linked cohort found ethnoracial disparities after recorded interpersonal violence [14]. Aggregate data cannot determine their contributions. Future regional analyses should incorporate race and ethnicity, socioeconomic characteristics, and health system indicators.

An immediate 23.31% level increase occurred in 2020, without a significant change in the subsequent slope. Because the interrupted time-series design cannot rule out concurrent changes in death reporting, cause-of-death coding, or population denominators during 2020, the level change should be interpreted as a temporal association with the pandemic period rather than evidence of a causal effect. Brazilian interrupted time-series studies reported different pandemic-associated patterns, including no national turning point and changes confined to subgroups [15-17]. International data from 33 countries or regions likewise showed no uniform increase during the first nine to 15 months of the pandemic [18]. These differences may reflect the populations studied and the specification of time and interruption. Earlier analyses included all ages, whereas the present study was restricted to people younger than 30 years. Because the trend remained significant after excluding the pandemic years, the 2020 increase appears to represent a discontinuity superimposed on an earlier rise rather than its origin.

Including deaths of undetermined intent and sequelae of intentional self-harm increased the estimated burden without materially changing the direction or timing of the trends. Analyses restricted to codes X60-X84 therefore underestimate absolute mortality. Redistribution of nonspecific external-cause codes can alter cause-specific mortality estimates in Brazil [8], and integration of mortality, hospitalization, and self-harm notification data may improve surveillance of fatal and nonfatal outcomes [19]. The differences identified across demographic groups and regions also support reporting disaggregated rather than national estimates alone.

Misclassification of intent in death certification is the principal limitation. The expanded definition reduced dependence on explicit self-harm coding but cannot determine which deaths of undetermined intent were suicides and may include deaths that were not. Concordance between definitions reduces, but does not eliminate, this uncertainty. The expanded definition may also have included deaths that were not self-inflicted, such as unintentional poisonings coded as undetermined intent. Completeness of death registration and cause-of-death coding practices improved unevenly over the study period and differed by region, which may affect comparability across years and across regions with historically lower registration coverage. The number of subgroup and joinpoint comparisons performed increases the likelihood that some strata-specific findings reflect chance rather than a true effect. As with the overall ecological design, the interrupted time-series analysis can describe an association with the pandemic period but cannot establish causation.

## Conclusions

Suicide mortality among Brazilians aged 10-29 years doubled from 2000 to 2023, with acceleration after 2015 and heterogeneous trends across age groups, sexes, and regions. National surveillance should routinely report narrow age groups and regional strata because aggregate estimates obscure these differences. Additional years of mortality data are needed to determine whether the recent declines among adolescents persist and to identify the factors underlying regional divergence.
